# Could lysosomal acid lipase enzyme activity be used for clinical follow-up in cryptogenic cirrhosis?

**DOI:** 10.55730/1300-0144.5410

**Published:** 2022-03-27

**Authors:** Engin KÖSE, Elçin ÇAĞATAY, Tutku YARAŞ, Pelin TEKE KISA, Seminay GÜLER, Zümrüt ARSLAN GÜLTEN, Mesut AKARSU, Yavuz OKTAY, Hülya AYAR KAYALI, Nur ARSLAN

**Affiliations:** 1 Department of Pediatric Metabolism, Faculty of Medicine, Ankara University, Ankara, Turkey; 2 Department of Molecular Biology and Genetics, International Biomedicine and Genome Institute, Dokuz Eylül University, İzmir, Turkey; 3 Department of Basic and Translational Research, International Biomedicine and Genome Center, İzmir, Turkey; 4 Department of Pediatric Metabolism, Faculty of Medicine, Dokuz Eylül University, İzmir, Turkey; 5 Department of Gastroenterology, Faculty of Medicine, Dokuz Eylül University, İzmir, Turkey; 6 Department of Medical Biology, Faculty of Medicine, Dokuz Eylül University, İzmir, Turkey; 7 Department of Science Chemistry, Dokuz Eylül University, İzmir, Turkey

**Keywords:** Cholesterol ester storage disease, cryptogenic cirrhosis, lysosomal acid lipase deficiency, *LIPA* gene

## Abstract

**Background/aim:**

Cholesterol ester storage disease (CESD) is one of the rare causes that should be kept in mind in the etiology of cirrhosis. Recent studies detected that significantly reduced lysosomal acid lipase deficiency enzyme (LAL) in patients with cryptogenic cirrhosis (CC). Moreover, studies have evaluated that LAL activity is as effective as scoring systems in assessing the severity of cirrhosis.

In this study, we aimed to investigate the CESD with LAL level and mutation analysis of *LIPA* gene in patients diagnosed with CC and to compare LAL activities between patients with CC and healthy volunteers.

**Materials and methods:**

Laboratory parameters and cirrhosis stage (CHILD and MELD) were recorded for the patient group included in the study. In addition, blood samples were taken from each case included in the study for LAL activity determination and *LIPA* gene analysis.

**Results:**

A statistically significant decrease in LAL activity was found in patients diagnosed with CC compared to the healthy group. *LIPA* gene analysis did not detect CESD in any patient group. Correlation analysis showed a positive correlation between LAL activity and white blood cell and platelet counts in both healthy volunteers and CC patient groups. In the univariate and multivariate logistic regression analysis of the parameters associated with the MELD of ≥10 in patients with CC, significant relationship was found between the MELD of ≥10 and the LAL activity.

**Conclusions:**

In our study, LAL activity was significantly lower in CC patients than in the normal population. LAL activity level appears to be a parameter that can be used to assess the severity of cirrhosis.

## 1. Introduction

Cholesterol ester storage disease (CESD) is a rare lysosomal storage disease characterized by a deficiency of the lysosomal acid lipase enzyme (LAL) activity due to a mutation in the *LIPA* gene. It is challenging to diagnose LAL deficiency in adulthood due to subtle symptoms and lack of a characteristic marker. On the other hand, a significant portion of adult patients with LAL deficiency shows asymptomatic liver enzyme elevation and presents with fatty liver disease, nonalcoholic steatohepatitis [1,2].

Patients developing cirrhosis due to cholesterol ester storage disease have been described in the literature [3,4]. Moreover, a recent study reported that the prevalence of LAL deficiency in unexplained liver diseases is 0.1% [5]. These findings indicate that patients with cryptogenic cirrhosis (CC) should be examined in terms of CESD.

In addition, although some studies have shown a decrease in LAL activity in patients with CC, no pathogenic variation has been detected in the LIPA gene. In a study conducted in 2016, it was found that LAL activity was decreased in patients with CC compared to the normal population [6]. Similarly, Baratta et al. detected lower LAL activity in cases with a diagnosis of CC compared to the normal population [7].

In this study, we aimed to analyze the level of LAL activity in CC patients and healthy volunteers, to detect LIPA gene mutations, and to compare the differences in LAL activity, clinical characteristics, and other laboratory parameters between these two groups.

## 2. Materials and methods

The study has been performed between October 2018 and October 2020. Thirty patients diagnosed with CC and 30 sex and age-matched healthy volunteers were enrolled in the study. Cryptogenic cirrhosis was defined as cirrhosis of unknown etiology in patients with a clinical history of previous overweight/obesity, diabetes, insulin resistance and/or liver steatosis, and no history of alcoholism or alcohol consumption higher than 20 g/d in men and 10 g/d in women, or previous acute or chronic viral and autoimmune hepatitis [8]. The severity of CC was assessed by the Child-Pugh score (CHILD) and Model for End-Stage Liver Disease score (MELD).

Total cholesterol, LDL-cholesterol, HDL-cholesterol, triglyceride, liver function tests, albumin, and cell blood count were assessed for all subjects. Abdominal imaging (ultrasound and magnetic resonance images) and echocardiographic findings of CC-diagnosed patients were evaluated.

### 2.1. Determination of lysosomal acid lipase enzyme activity by DBS method

The LAL activity was measured by optimizing the method reported by Dairaku et al. [9]. In brief, 3 mm spots were eluted in 200 μL of sterile water for 1 h at room temperature. Inhibited and uninhibited reactions were carried out in 3 replicates for each patient. For inhibited reactions, 40 μL of blood samples and 10 μL of sterile water were added to each well. In contrast, for uninhibited reactions, 40 μL of blood samples and 10 μL of Lalistat 2 were added to each well, and the total volume in the wells was completed to 100 μL with substrate buffer. Plates were covered with adhesive aluminum film and incubated at 37 °C for 24 h. The reaction was terminated by adding 200 μL of 150 mM EDTA in the wells. Fluorescence intensity was measured using the Varioscan Thermo device (Ex/Em: 320/450 nm).

### 2.2. LIPA gene analysis

#### 2.2.1. DNA isolation protocol

Blood samples were collected in EDTA tubes, aliquoted, and stored at −80 °C until DNA isolation. For DNA isolation, 1 mL blood was mixed with 10 mL Buffer A (10 mM Tris-HCl, 320 mM sucrose, 5 MgCl_2_, 1% Triton X-100, pH 8.0) for 4 min at room temperature. Cells were pelleted by centrifugation at 3000 rpm for 10 min. Pellet was resuspended in 5 mL Buffer A and repelleted by centrifugation at 3000 rpm for 10 min. Two mL Buffer B (400 mM Tris-HCl, 0.5 M EDTA, 150 mM NaCl, 1% SDS) was added to the pellet, and cells were lysed by pipetting. Five hundred μL sodium perchlorate (NaCl_4_) was added and further incubated for 10 min on a shaker at room temperature. Next, it was incubated at 65 °C for 25 min, with vortexing every 5 min. Two mL chloroform (at −20 °C) was added and incubated for 10 min on a shaker. After centrifugation at 3000 rpm for 10 min at +4 °C, the supernatant was transferred to a new tube. Six mL EtOH (100%) was added and mixed until DNA precipitate was visible as “medusa”, which was next transferred to an Eppendorf tube with 500 μL EtOH (70%). DNA was pelleted by centrifugation at 14000 rpm for 1 min and air-dried. DNA was resuspended in 50–100 μL of 5 mM Tris-EDTA pH 8.0 and left at +4 °C overnight. The next day, it was incubated at 55 °C for 1 h and stored at −20 °C.

#### 2.2.2. PCR amplification of LIPA exons from genomic DNA

All ten exons of the LIPA gene (Major transcript LIPA-202, NM_000234.4, GRCh38.p13) were amplified from genomic DNA. Primers were designed using NCBI’s Primer-BLAST tool.

#### 2.2.3. Sanger sequencing and variant interpretation

PCR products were cleaned up by ExoSAP-IT, and sequencing PCR was performed using the BigDye Terminator Kit. Sequencing analysis was performed by using the ABI 3730 × l DNA Analyzer. Chromatograms were manually checked using SnapGene 5.2 and aligned to the reference human genome (GRCh38.p13) using NCBI-BLAST (BLASTN) tool. Potential pathogenicity of identified variants was assessed with the help of databases, including gnomAD, ClinVar, Varsome, and dbSNP, according to the ACMG guidelines.

### 2.3. Statistical analysis

The categorical variable (gender) was assessed using chi-square test and expressed as number and percentage. The Shapiro-Wilk tests were performed to evaluate the normality of continuous variables. All continuous variables were expressed as mean ± SD (min-max) and median. Nonparametric continuous variables were analyzed with the Mann-Whitney U test, while parametric continuous variables were assessed with a t-test. Pearson’s correlation coefficient was used to analyze the associations between laboratory parameters. The relationship between laboratory parameters and CC and the MELD score of ≥10 was analyzed by logistic regression. Receiver operator characteristic (ROC) curve analysis was used to determine threshold values in the laboratory to establish LAL activity to differentiate CC and MELD score of ≥10 in patients with CC. Specificity, sensitivity, and positive and negative predictive value of LAL activity were determined.

Data were analyzed with the Statistical Package for Social Sciences (SPSS) software (version 21.0; SPSS, Chicago, IL, USA). A two-tailed p-value < 0.05 was considered significant.

## 3. Results

Thirty patients with the diagnosis of CC and sex and age-matched healthy subjects were enrolled in the study. The mean age of patients with CC was 53.3 ± 9.9 (28–75) years. In physical examination, gastrointestinal tract endoscopy, abdominal ultrasonography, and MRI, esophageal varices were detected in 26 (86.7%) patients, splenomegaly was detected in 23 (76.7%), and hepatomegaly was seen in 10 (33.3%) patients. Hepatosteatosis and hepatic fibrosis were detected in 6 (20%) and 3 (10%) patients, respectively (Table 1).

A pathogenic variant in the *LIPA* gene was not found in any of the cases. In addition, there was no relationship between the presence of benign variants and LAL activity. In the assessment of laboratory parameters, higher ALT, AST, GGT, ALP, total bilirubin, direct bilirubin, INR, and aPTT levels were found in CC diagnosed patients compared to healthy subjects. Serum albumin, total cholesterol, LDL-C and HDL-C levels, and WBC, hemoglobulin, and platelet counts were found to be lower in CC diagnosed patients compared to healthy subjects. There were no differences between the two groups in terms of serum triglyceride HDL-C levels and MPV (Table 1). Statistically significantly reduced LAL activity was found in CC diagnosed patients compared to the healthy group (Table 1).

Correlation analysis showed positive correlations between LAL activity and WBC (r = 0.677, p < 0.0001) and platelet counts (r = 0.566, p = 0.001) in healthy subjects (Figure a, b). Positive correlations between LAL activity and WBC (r = 0.527, p = 0.003) and platelet counts (r = 0.599, p < 0.0001) were detected in patients with CC (Figure c, d). In patients with CC, a statistically significant negative correlation between MELD score and LAL activity was revealed (r = −0.409, p = 0.025) (Figure e).

Univariate and multivariate logistic regression analysis was performed to determine parameters associated with CC. Associations between CC and WBC [OR: 0.998 (0.996–1.000), p = 0.034], albumin [OR: 0.0001 (0.0001–0.370), p = 0.038] and INR [8.257 × 10^18^ (3.498–1.949 × 10^37^, p = 0.044)] were determined (Table 2).

Univariate and multivariate logistic regression analysis showed that INR [245165746 (7.365–8.161 × 10^15^), p=0.029] and LAL activity [0.0001 (0.0001–0345), p = 0.034] were associated with the MELD score of ≥10 in patients with CC (Table 3).

The diagnostic value of LAL activity for the prediction of CC was assessed with ROC analysis. Lysosomal acid lipase DBS activity ≤0.419 nmol/spot/h was almost identical with regard to best prediction of CC. Sensitivity and specificity of LAL activity were 70% (50.6%–80.3%) and 66.7% (47.2%–82.7%), respectively (Table 4).

ROC analysis was performed to calculate the diagnostic value of LAL activity for the prediction of MELD score ≥10 in patients with CC. Lysosomal acid lipase enzyme activity ≤0.396 nmol/spot/h was almost identical with regard to best prediction of MELD score ≥10 in patients with CC. Sensitivity and specificity of LAL activity for prediction of MELD score ≥10 was determined as 86.7% (59.5%–98.3%) and 60.0% (32.3%–83.7%), respectively (Table 5).

## 4. Discussion

Our study is one of the rare studies investigating the relationship between LAL activity and CC and the possible presence of CESD in patients with CC. The most important finding of this study is that the patients with CC have lower LAL activity compared to the healthy population. Due to the detection of no pathogenic variation of the *LIPA* gene, the lower LAL activity of CC diagnosed patients was not associated with cholesterol ester storage disease. Consistently, a study determined reduced LAL activity in patients with CC relative to the healthy subjects [6]. Moreover, in a study involving 60 patients with CC and 100 healthy volunteers, Baratta et al. reported that CC diagnosed patients have lower LAL activity compared to the healthy population [7].

As expected, most of the LAL activity is dosed in DBS derives from the leukocyte, and it is suggested that the low leukocyte count detected in cirrhosis patients leads to a decrease in LAL activity [6,10]. In our study, a positive significant correlation was detected between LAL activity and WBC in both groups. Interestingly, the same relationship between enzyme activity and platelets was determined in both the previous and our study [6]. Although thrombocytes are known to contain lysosomes, it is thought that contribution of lysosomal enzyme activity in thrombocytes is restricted in the DBS method. Thrombocytopenia is associated with the severity of cirrhosis, and severe cirrhosis may cause low LAL activity. However, this hypothesis cannot explain the relationship between enzyme activity and platelets in a healthy group.

Cholesterol ester storage disease is one of the rare causes that should be kept in mind in the etiology of cirrhosis. According to a recent study, the prevalence of LAL deficiency in unexplained liver diseases was reported as 0.1% [5]. Previously, cholesterol ester storage disease has been described as an etiology of cirrhosis [3,4]. Although low LAL activity creates confusion in the possible underlying cholesterol ester storage disease in cirrhosis patients of unknown etiology, these patients should be genetically evaluated for LAL deficiency. In our study, no diagnosis of cholesterol ester storage disease was made in any patient with CC. In our opinion, the small size of our study group and the fact that our study was conducted in a single-center caused this situation. We believe that multicenter studies with larger number of patients are needed to determine the prevalence of cholesterol ester storage disease in cirrhosis patients of unknown etiology.

Not surprisingly, in cirrhosis patients, deterioration in liver function tests, decreased leukocyte and platelet count, and impaired bleeding profile are expected laboratory findings. In this respect, a significant difference was found between the two groups when compared with the healthy control group. In the analysis of the relationship between laboratory findings and LAL activity, our study revealed a significant positive correlation with both WBC and platelet counts. Likewise, Vespasiani-Gentilucci et al. detected a positive correlation between platelet count and LAL activity in healthy volunteers. Furthermore, they determined a positive correlation between enzyme activity and WBC and platelet counts in CC diagnosed patients [6]. Similarly, in another study, a significant positive correlation was found between LAL activity and white blood cell and platelet count [11].

There are different scoring systems (CHILD, MELD) used to determine the severity of cirrhosis [12]. Several studies have evaluated whether LAL activity is as effective as these scoring systems in assessing the severity of cirrhosis, and contrasting results have been reported [11,13,14]. While a publication revealed no relationship between LAL activity and cirrhosis severity [14], a weak significant association was found in some studies [11,13]. In the study conducted by Angelico et al., a weakly significant correlation was found between the CHILD and MELD scoring and LAL activity [11]. We detected a significant positive correlation between the MELD score and LAL activity. To date, prediction of MELD score ≥10 with LAL activity had not been evaluated in literature. LAL activity was found to have 87% sensitivity and 60% specificity in predicting patients with a MELD score of 10 and above. These findings suggest that LAL activity may be effective in determining the severity of cirrhosis.

The effectiveness of LAL enzyme activity as a parameter to predict CC has been evaluated in a recent study [14]. Gravito-Soares et al. stated that LAL activity could be used in the diagnosis of CC disease. It was determined that LAL activity could diagnose CC with 86.2% sensitivity and 75% specificity [14]. Although the values in our study are not as high as reported, it was calculated that LAL activity predicted the diagnosis of CC with 70% sensitivity and 67% specificity.

In this study, the small number of subgroups and the lack of a prospective follow-up period limited the evaluation of the effectiveness of LAL activity in predicting CC and determining the severity of CC.

In conclusion, LAL activity was found to be significantly lower in CC patients compared to the healthy population. The level of LAL activity appears to be a parameter that can be used to assess the severity of cirrhosis. Although CESD was not detected by genetic analysis in any of our patients with CC, the cases reported in the literature suggest that CESD should be investigated in patients with CC. Future longitudinal prospective studies with larger sample sizes are needed to confirm our findings.

## Figures and Tables

**Figure:**
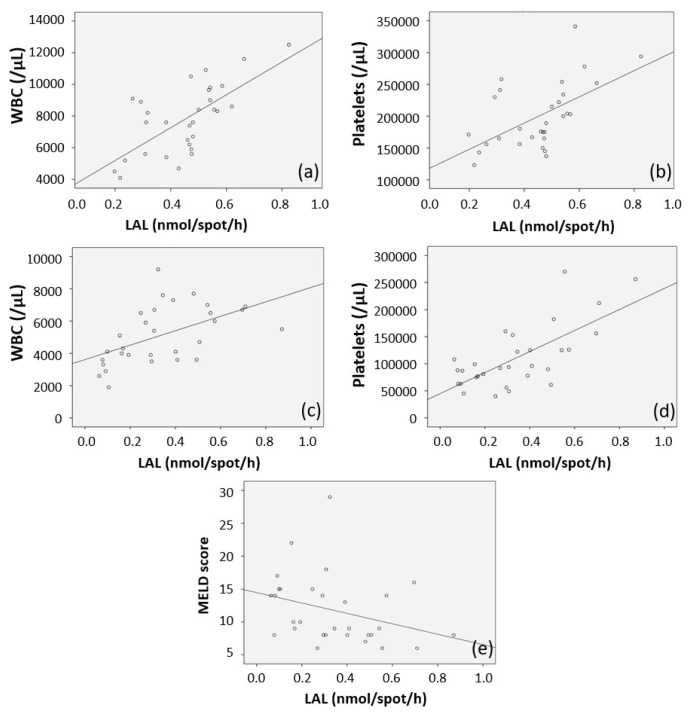
Correlation analysis of laboratory parameters in healthy group (a) LAL (nmol/spot/h) level and WBC (r = 0.677, p < 0.0001), (b) LAL (nmol/spot/h) and platelets (r = 0.566, p = 0.001). Correlation analysis of laboratory parameters in patients with CC (c) LAL (nmol/spot/h) level and WBC (r = 0.527, p = 0.003), (d) LAL (nmol/spot/h) and platelets (r = 0.599, p < 0.0001), (e) LAL (nmol/spot/h) level and MELD score (r = −0.409, p = 0.025) in patients with CC.

**Table 1:** Demographic, clinic and laboratory findings of healthy control group and patients with CC.

Parameters	Healthy group (n = 30)	Cryptogenic cirrhosis (n = 30)	p

**Age (year)**			
**mean ± SD (min-max)**	52.9 ± 4.9 (47–63)	53.3 ± 9.9 (28–75)	0.397†
**median**	52.0	54.5	

**Gender (F/M), n (%)**	14 (46.7)/16 (53.3)	14 (46.7)/16 (53.3)	1.000

**Esophageal varices, n (%)**	0 (0)	26 (86.7)	

**Splenomegaly, n (%)**	0 (0)	23 (76.7)	

**Hepatomegaly, n (%)**	0 (0)	10 (33.3)	

**Intraabdominal acid, n (%)**	0 (0)	9 (30.0)	

**Hepatosteatosis, n (%)**	0 (0)	6 (20.0)	

**Hepatic fibrosis, n (%)**	0 (0)	3 (10.0)	

**CHILD (a/b/c), n (%)**	-	21(70.0)/2(6.7)/7(23.3)	

**ALT (IU/L)**			
**mean ± SD (min-max)**	20.2 ± 7.9 (15–29)	28.2 ± 14.5 (9–65)	<0.0001†
**median**	20.5	25.0	

**AST (IU/L)**			
**mean ± SD (min-max)**	20.6 ± 3.9 (15–29)	41.9 ± 25.8 (19–139)	<0.0001†
**median**	20.5	31.5	

**GGT (IU/L)**			
**mean ± SD (min-max)**	21.2 ± 8.2 (11–44)	58.7 ± 34.2 (13–170)	<0.0001†
**median**	21.0	52.5	

**ALP (IU/L)**			
**mean ± SD (min-max)**	79.7 ± 19.0 (45–110)	121.4 ± 75.2 (53–429)	0.005†
**median**	79.0	104.5	

**Albumin (mg/dL)**			
**mean ± SD (min-max)**	4.3 ± 0.2 (3.9–4.6)	3.6 ± 0.6 (2.1–4.6)	<0.0001‡
**median**	4.4	3.7	

**Total bilirubin (mg/dL)**			
**mean ± SD (min-max)**	0.7 ± 0.2 (0.4–1.1)	2.2 ± 2.3 (0.4–12.1)	<0.0001†
**median**	0.6	1.35	

**Direct bilirubin (mg/dL)**			
**mean ± SD (min-max)**	0.09 ± 0.04 (0.01–0.2)	0.7 ± 1.3 (0.01–7)	<0.0001†
**median**	0.09	2.8	

**TC (mg/dL)**			
**mean ± SD (min-max)**	212.1 ± 33.7 (138.0–298.0)	176.3 ± 39.0 (93.0–299.0)	<0.0001†
**median**	204.0	172.0	

**LDL-C (mg/dL)**			
**mean ± SD (min-max)**	134.2 ± 24.7 (77.0–191.0)	111.2 ± 33.1 (51.0–200.0)	0.002†
**median**	134.5	107.0	

**HDL-C (mg/dL)**			
**mean ± SD (min-max)**	50.1 ± 14.5 (28.0–87.0)	42.6 ± 13.3 (23.0–73.0)	0.058†
**median**	45.0	42.0	

**Triglyceride (mg/dL)**			
**mean ± SD (min-max)**	116.9 ± 56.6 (57–245)	111.4 ± 70.5 (23–370)	0.549†
**median**	98.5	98.5	

**WBC (10** **^3^** **/μL)**			
**mean ± SD (min-max)**	7.8 ± 2.2 (4.1–12.5)	5.1 ± 1.8 (1.9–9.2)	<0.0001‡
**median**	7.9	4.9	

**Hemoglobin (g/dL)**			
**mean ± SD (min-max)**	13.7 ± 1.4 (11.6–16.4)	11.8 ± 2.2 (7.6–15.8)	<0.0001‡
**median**	13.9	11.9	

**Platelets (10** **^3^** **/μL)**			
**mean ± SD (min-max)**	199.9 ± 51.7 (123–341)	110.9 ± 58.4 (40–270)	<0.0001†
**median**	184.5	93.0	

**MPV (fL)**			
**mean ± SD (min-max)**	8.7 ± 1.1 (6.7–11.4)	9.0 ± 0.9 (6.6–11.2)	0.325‡
**median**	8.6	8.9	

**INR**			
**mean ± SD (min-max)**	0.91 ± 0.09 (0.78–1.20)	1.22 ± 0.26 (0.90–2.00)	<0.0001†
**median**	0.9	1.2	

**aPTT (second)**			
**mean ± SD (min-max)**	29.2 ± 2.2 (25.5–33.3)	36.3 ± 7.1 (25.9–54.8)	<0.0001†
**median**	28.9	35.0	

**LAL activity (nmol/spot/h)**			
**mean ± SD (min-max)**	0.45 ± 0.14 (0.19–0.82)	0.34 ± 0.21 (0.06–0.87)	0.019‡
**median**	0.47	0.30	

† Mann Whitney U test,

‡ student’s t-test,

ALT: alanine aminotransferase, ALP: alkaline phosphatase, aPTT: activated partial thromboplastin time, AST: aspartate aminotransferase, GGT: gamma glutamyl transferase, HDL-C: high-density lipoprotein cholesterol, INR: international normalized ratio, LAL: lysosomal acid lipase enzyme, LDL-C: low-density lipoprotein cholesterol, min: minimum, max: maximum, MPV: mean platelet volume, SD: standard deviation, TC: total cholesterol, WBC: white blood cell.

**Table 2:** Univariate and multivariate logistic regression analysis of parameters related with CC.

Parameters	Univariate regression model		Multivariate regression model	
	OR (95% Cl Lower-upper)	p	OR (95% Cl Lower-upper)	p
**Age (year)**	1.007 (0.943–1.075)	0.841		
**LAL activity (nmol/spot/h)**	0.29 (0.001–0.629)	0.024		
**WBC (10** **^3^** **/μL)**	0.999 (0.999–1.000)	<0.0001	0.998 (0.996–1.000)	0.034
**Platelets (10** **^3^** **/μL)**	1.000 (1.000–1.000)	<0.0001		
**ALT (IU/L)**	1.069 (1.010–1.130)	0.02		
**AST (IU/L)**	1.346 (1.149–1.575)	<0.0001		
**GGT (IU/L)**	1.133 (1.060–1.211)	<0.0001		
**ALP (IU/L)**	1.032 (1.009–1.055)	0.006		
**Albumin (mg/dL)**	0.001 (0.0001–0.037)	<0.0001	0.0001 (0.0001–0.370)	0.038
**Total bilirubin (mg/dL)**	8979.518 (19.521–39625.945)	<0.0001		
**Direct bilirubin (mg/dL)**	2.066 × 10^9^ (13354.383–3.195 × 10^14^)	<0.0001		
**TC (mg/dL)**	0.971 (0.954–0.989)	0.002		
**LDL-C (mg/dL)**	0.973 (0.954–0.992)	0.007		
**HDL-C (mg/dL)**	0.960 (0.922–1.000)	0.049		
**Triglyceride (mg/dL)**	0.999 (0.954–1.007)	0.732		
**Hemoglobin (g/dL)**	0.558 (0.391–0.796)	0.001		
**MPV (fL)**	1.295 (0.778–2.154)	0.320		
**INR**	3691465.64 (2051.491–6.642 × 10^9^)	<0.0001	8.257 × 10^18^ (3.498–1.949 × 10^37^)	0.044
**aPTT (second)**	1.538 (1.224–1.932)	<0.0001		

ALT: alanine aminotransferase, ALP: alkaline phosphatase, aPTT: activated partial thromboplastin time, AST: aspartate aminotransferase, Cl: confidence intervals, GGT: gamma glutamyl transferase, HDL-C: high-density lipoprotein cholesterol, INR: international normalized ratio, LAL: lysosomal acid lipase enzyme, LDL-C: low-density lipoprotein cholesterol, MPV: mean platelet volume, OR: odds ratio TC: total cholesterol, WBC: white blood cell.

**Table 3:** Univariate and multivariate logistic regression analysis of parameters related with MELD score ≥10 in patients with CC.

Parameters	Univariate regression model		Multivariate regression model	
	OR (95% Cl Lower-upper)	p	OR (95% Cl Lower-upper)	p
**Age (year)**	0.936 (0.857–1.023)	0.146		
**LAL activity (nmol/spot/h)**	0.007 (0.0001–0.656)	0.032	0.0001 (0.0001–0345)	0.034
**WBC (10** **^3^** **/μL)**	1.000 (0.999–1.000)	0.549		
**Platelets (10** **^3^** **/μL)**	1.000 (1.000–1.000)	0.098		
**ALT (IU/L)**	1.003 (0.954–1.055)	0.899		
**AST (IU/L)**	1.039 (0.994–1.086)	0.089		
**GGT (IU/L)**	1.010 (0.987–1.033)	0.899		
**ALP (IU/L)**	1.009 (0.995–1.024)	0.206		
**Albumin (mg/dL)**	0.101 (0.016–0.628)	0.014		
**Total bilirubin (mg/dL)**	332.980 (1.931–57412.384)	0.027		
**Direct bilirubin (mg/dL)**	3861146.34 (2.700–5.522 × 10^12^)	0.036		
**TC (mg/dL)**	0.993 (0.974–1.013)	0.480		
**LDL-C (mg/dL)**	0.998 (0.976–1.020)	0.862		
**HDL-C (mg/dL)**	1.016 (0.961–1.074)	0.588		
**Triglyceride (mg/dL)**	0.992 (0.980–1.005)	0.247		
**Hemoglobin (g/dL)**	0.675 (0.453–1.007)	0.054		
**MPV (fL)**	1.153 (0.548–2.425)	0.707		
**INR**	130315.050 (14.607–1.163 × 10^9^)	0.011	245165746 (7.365–8.161 × 10^15^)	0.029
**aPTT (second)**	1.116 (0.984–1.266)	0.087		

ALT: alanine aminotransferase, ALP: alkaline phosphatase, aPTT: activated partial thromboplastin time, AST: aspartate aminotransferase, Cl: confidence intervals GGT: gamma glutamyl transferase, HDL-C: high-density lipoprotein cholesterol, INR: international normalized ratio, LAL: lysosomal acid lipase enzyme, LDL-C: low-density lipoprotein cholesterol, MPV: mean platelet volume, OR: odds ratio TC: total cholesterol, WBC: white blood cell.

**Table 4:** Diagnostic value and ROC analysis of LAL enzyme activity for prediction of CC.

Parameters	Sensitivity (%) (95% Cl)	Specificity (%) (95% Cl)	PPV (%) (95% Cl)	NPV (%) (95% Cl)	Accuracy (%) (95% Cl)	ROC analysis	
						AUC (95% Cl)	p
**LAL activity (nmol/spot/h) ≤ 0.419 nmol/spot/h**	70.0 (50.6–80.3)	66.7 (47.2–82.7)	67.7 (54.6–78.6)	68.9 (54.9–80.2)	68.3 (55.0–79.7)	0.677 (0.537–0.817)	0.019

AUC: area under curve, Cl: confidence intervals, LAL: lysosomal acid lipase, NPV: negative predictive value, PPV: positive predictive value, ROC: receiver operating characteristic.

**Table 5:** Diagnostic value and ROC analysis of LAL enzyme activity for prediction of MELD score ≥10 in patients with CC.

Parameters	Sensitivity (%) (95% Cl)	Specificity (%) (95% Cl)	PPV (%) (95% Cl)	NPV (%) (95% Cl)	Accuracy (%) (95% Cl)	ROC analysis	
						AUC (95% Cl)	p
**LAL activity (nmol/spot/h) ≤ 0.396 nmol/spot/h**	86.7 (59.5–98.3)	60.0 (32.3–83.7)	68.42 (53.1–80.6)	81.8 (53.7–87.7)	73.3 (54.1–87.7)	0.756 (0.573–0.938)	0.017

AUC: area under curve, Cl: confidence intervals, LAL: lysosomal acid lipase enzyme, NPV: negative predictive value, PPV: positive predictive value, ROC: receiver operating characteristic.

## References

[1] Tovoli F Napoli L Negrini G D’Addato S Tozzi G A Relative Deficiency of Lysosomal Acid Lypase Activity Characterizes Non-Alcoholic Fatty Liver Disease International Journal of Molecular Sciences 2017 18 6 1134 10.3390/ijms18061134 28587063 PMC5485958

[2] Selvakumar PK Kabbany MN Lopez R Tozzi G Alisi A Reduced lysosomal acid lipase activity - A potential role in the pathogenesis of non alcoholic fatty liver disease in pediatric patients Digestive and Liver Disease 2016 48 8 909 913 10.1016/j.dld.2016.04.014 27198736

[3] Pant M Oshima K Cholesteryl Ester Storage Disease: An underdiagnosed cause of cirrhosis in adults Annals of Diagnostic Pathology 2017 31 66 70 10.1016/j.anndiagpath.2017.02.005 28318950

[4] Reynolds T Cholesteryl ester storage disease: a rare and possibly treatable cause of premature vascular disease and cirrhosis Journal Of Clinical Pathology 2013 66 11 918 923 10.1136/jclinpath-2012-201302 23999269

[5] Kuloglu Z Kansu A Selbuz S Kalaycı AG Şahin G National LAL-D Study Group The Frequency of Lysosomal Acid Lipase Deficiency in Children With Unexplained Liver Disease Journal of Pediatric Gastroenterology and Nutrition 2019 68 3 371 376 10.1097/MPG.0000000000002224 30540705

[6] Vespasiani-Gentilucci U Gallo P Piemonte F Riva E Porcari A Lysosomal Acid Lipase Activity Is Reduced Both in Cryptogenic Cirrhosis and in Cirrhosis of Known Etiology PLoS One 2016 11 5 e0156113 10.1371/journal.pone.0156113 27219619 PMC4878774

[7] Baratta F Pastori D Tozzi G D’Erasmo L Di Costanzo A Lysosomal acid lipase activity and liver fibrosis in the clinical continuum of non-alcoholic fatty liver disease Liver International 2019 39 12 2301 2308 10.1111/liv.14206 31392821

[8] Caldwell SH Oelsner DH Iezzoni JC Hespenheide EE Battle EH Cryptogenic cirrhosis: clinical characterization and risk factors for underlying disease Hepatology 1999 29 3 664 669 10.1002/hep.510290347 10051466

[9] Dairaku T Iwamoto T Nishimura M Endo M Ohashi T A practical fluorometric assay method to measure lysosomal acid lipase activity in dried blood spots for the screening of cholesteryl ester storage disease and Wolman disease Molecular Genetics and Metabolism 2014 111 2 193 196 10.1016/j.ymgme.2013.11.003 24295952

[10] Ceci R Francesco P Mucci J Cancelarich L Fossati C Reliability of enzyme assays in dried blood spots for diagnosis of 4 lysosomal storage disorders Advances in Biological Chemistry 2011 1 58 64 10.4236/abc.2011.13008

[11] Angelico F Corradini SG Pastori D Fargion S Fracanzani AL LAL-Cirrhosis Collaborative Research Group Severe reduction of blood lysosomal acid lipase activity in cryptogenic cirrhosis: A nationwide multicentre cohort study Atherosclerosis 2017 262 179 184 10.1016/j.atherosclerosis.2017.03.038 28396038

[12] Papatheodoridis GV Cholongitas E Dimitriadou E Touloumi G Sevastianos V MELD vs Child-Pugh and creatinine-modified Child-Pugh score for predicting survival in patients with decompensated cirrhosis World Journal of Gastroenterology 2005 11 20 3099 3104 10.3748/wjg.v11.i20.3099 15918197 PMC4305847

[13] Shteyer E Villenchik R Mahamid M Nator N Safadi R Low Serum Lysosomal Acid Lipase Activity Correlates with Advanced International Journal of Molecular Sciences 2016 17 3 312 10.3390/ijms17030312 26927097 PMC4813175

[14] Gravito-Soares M Gravito-Soares E Gomes D Tome L Lysosomal Acid Lipase: Can it be a New Non-Invasive Serum Biomarker of Cryptogenic Liver Fibrosis and Cirrhosis? Annals of Hepatology 2019 18 1 78 88 10.5604/01.3001.0012.7865 31113613

